# Maternal diabetes amplifies the influence of maternal asthma and smoke exposure on the development of asthma in offspring

**DOI:** 10.1186/1710-1492-7-S2-A22

**Published:** 2011-11-14

**Authors:** Meghan B Azad, Allan B Becker, Anita L Kozyrskyj

**Affiliations:** 1 Women and Children’s Health Research Institute, Department of Pediatrics, University of Alberta, Edmonton, Canada; 2 Department of Pediatrics and Child Health, University of Manitoba, Winnipeg, Canada

## Background

Perinatal programming is an emerging theory for the fetal origins of chronic disease. Maternal asthma and environmental tobacco smoke (ETS) are two of the best-known triggers for the perinatal programming of asthma, while the potential role of maternal diabetes has not been widely studied. The goal of this study was to determine if maternal diabetes contributes to the perinatal programming of asthma, and if so, whether its effect is additive or synergistic with respect to ETS exposure and maternal asthma.

## Methods

We studied 3,574 Canadian children, aged 7-8 yr, enrolled in a population-based birth cohort. Standardized questionnaires were completed by the children’s parents, and data were analyzed by multivariate logistic regression.

## Results

Asthma was reported in 442 children (12.4%). Asthmatic children were more likely to have mothers, but not fathers, with diabetes. In children without maternal history of diabetes, ETS exposure increased the risk of child asthma by 1.4-fold (adjusted odds ratio, 1.40; 95% confidence interval, 1.13-1.73), while maternal asthma increased risk by 3.6-fold (3.59; 2.71-4.76). In children born to diabetic mothers, these effects were amplified to 5.7-fold (5.68; 1.18-27.37) and 11.3-fold (11.30; 2.26-56.38), respectively. There was no independent effect of maternal diabetes after adjusting for maternal asthma and ETS exposure (OR 0.65, 95%CI 0.16-2.56).

## Conclusions

Maternal diabetes contributes to the perinatal programming of child asthma by amplifying the detrimental effects of ETS exposure and maternal asthma.

